# Self-rated worry is associated with hospital admission in out-of-hours telephone triage – a prospective cohort study

**DOI:** 10.1186/s13049-020-00743-8

**Published:** 2020-06-10

**Authors:** Hejdi Gamst-Jensen, Erika Frischknecht Christensen, Freddy Lippert, Fredrik Folke, Ingrid Egerod, Linda Huibers, Mikkel Brabrand, Janne Schurmann Tolstrup, Lau Caspar Thygesen

**Affiliations:** 1 Emergency Medical Services Copenhagen, Copenhagen University, Copenhagen, Denmark; 2 Clinical Research Centre, Amager and Hvidovre Hospital, University of Copenhagen, Copenhagen, Denmark; 3 Clinic of Internal and Emergency Medicine and Department of Anesthesiology and Intensive Care, Aalborg University Hospital, Aalborg, Denmark; 4 Center for Prehospital and Emergency Research, Department of Clinical Medicine, Aalborg University, Aalborg, Denmark; 5 Department of Cardiology, Gentofte Hospital, University of Copenhagen University Hospital, Copenhagen, Denmark; 6 Department of Intensive Care, Rigshospitalet, University of Copenhagen, Copenhagen, Denmark; 7 Research Unit for General Practice, Aarhus, Denmark; 8 Department of Emergency Medicine, Odense University Hospital, Odense, Denmark; 9 Department of Emergency Medicine, Hospital of South West Jutland, Esbjerg, Denmark; 10 National Institute of Public Health, University of Southern Denmark, Copenhagen, Denmark

**Keywords:** Decision support systems, Emergency medicine, Telephone hotlines, Triage, Patient-centered care, Decision making, Help-seeking behavior

## Abstract

**Objective:**

Telephone triage manages patient flow in acute care, but a lack of visual cues and vague descriptions of symptoms challenges clinical decision making. We aim to investigate the association between the caller’s subjective perception of illness severity expressed as “degree-of-worry” (DOW) and hospital admissions within 48 h.

**Design and setting:**

A prospective cohort study was performed from January 24th to February 9th, 2017 at the Medical Helpline 1813 (MH1813) in Copenhagen, Denmark. The MH1813 is a primary care out-of-hours service.

**Participants:**

Of 38,787 calls received at the MH1813, 11,338 met the inclusion criteria (caller being patient or close friend/relative and agreement to participate). Participants rated their DOW on a 5-point scale (1 = minimum worry, 5 = maximum worry) before talking to a call handler.

**Main outcome measure:**

Information on hospitalization within 48 h after the call, was obtained from the Danish National Patient Register. The association was assessed using logistic regression in three models: 1) crude, 2) age-and-gender adjusted and 3) age, gender, co-morbidity, reason for calling and caller status adjusted.

**Results:**

A total of 581 participants (5.1%) were admitted to the hospital, of whom 170 (11.3%) presented with a maximum DOW, with a crude odds ratio (OR) for hospitalization of 6.1 (95% confidence interval (CI) 3.9 to 9.6) compared to minimum DOW. Estimates showed dose-response relationship between DOW and hospitalization. In the fully adjusted model, the ORs decreased to 3.1 (95%CI 2.0 to 5.0) for DOW = 5, 3.2 (2.0 to 5.0) for DOW = 4, 1.6 (1.0 to 2.6) for DOW = 3 and 0.8 (0.5 to 1.4) for DOW = 2 compared to minimum DOW.

**Conclusion:**

Patients’ self-assessment of illness severity as DOW was associated with subsequent hospital admission. Further, it may be beneficial in supporting clinical decision making in telephone triage. Finally, it might be useful as a measure to facilitate patient participation in the triage process.

## Background

Telephone triage is widely used in acute care and emergency medicine. It encompasses both emergency calls and non-life-threatening acute situations. Telephone triage is used to determine urgency and the type of health care needed and also to manage patient flow, but it is a difficult task due to the lack of visual cues and the innate gate-keeping role of the service [1–3]. Most calls to medical helplines are for non-life-threatening conditions, often presented with an array of symptoms that might not fit textbook descriptions [4, 5]. Nevertheless, conditions with a potentially severe outcome are also part of the incoming calls and it is crucial to identify these calls. Triage tools are recommended to aid health care professionals’ decision making [6], but these tools generally perform well only in the low and high levels of urgency, but less so in the middle triage categories [6, 7], moreover, they are criticized for not incorporating the patient’s context and perception nearly enough [8, 9]. In a Danish study from 2019 based on 200 calls to a medical helpline, researchers found that less than 2% of the callers were invited to express their emotional state [10]. Therefore, a need exists for a tool that systematically incorporates the patient’s perception of the situation and symptoms in telephone triage.

People tend to form their own perception of the situation in case of an illness or injury [11]. This is exemplified by the Common Sense Model showing five cognitive dimensions of illness representation: *identity* – the label the person assigns to the symptoms; *consequences* – the expected outcome of the symptoms; *cause* – idea of what caused the illness; *timeline* – expected duration of the illness, and perceived *cure or control* over the disease [12]. Moreover, three emotional representations (i.e. fear, anger and distress) incorporate the negative reactions to illness [11, 13]. Emotional representations, such as concern, have been found to predict worse illness outcome [14]. Worry, or concern, is the most frequent motivator for seeking help out-of-hours, and studies of malpractice claims in telephone triage show, that a failure to listen to the patient and the use of closed questions compromise patient safety [15–17]. The newly developed degree-of-worry scale (DOW), which was tested on 180 callers at a medical helpline, showed promising results to aid patient centered communication, in the regard that patients volunteered more medical-and-context-related information [18]. Moreover, self-rated health has been recognized as a valid predictor of all-cause mortality for decades. People with poor self-rated health status have approximately twice as high all-cause mortality than those reporting good health [19–21]. Self-rated health is an inclusive, usually five-point rating, that is thought to provide individual but contextual relevant information on bodily conditions, sensations, emotions and feelings [22, 23]. The DOW scale provides the call handler with the callers’ perception of illness severity measured as DOW. Considering that people can predict mortality through self-rated health, it might be valid to include DOW as an addition to current triage tools.

We aimed to investigate the association between the caller’s subjective perception of illness severity expressed as “degree-of-worry” (DOW) and the likelihood of being admitted to the hospital within 48 h. We used hospital admission within 48 h following a call to a medical helpline as a proxy of illness severity [6].

## Methods

### Study design

A prospective cohort study that combines the callers self-reported worry (DOW) with data from nationwide registers to evaluate the association with hospital admission [24].

### Setting

The Capital Region of Denmark covers an area of 2568 km^2^ with a population of 1.8 million people [25]. The acute care system within the Capital Region of Denmark, Copenhagen, offers two different access points (telephone numbers) to the Emergency Medical Service: 1–1-2 for the presumed life-threatening injuries and illnesses and the medical helpline 1813 (MH1813) for non-life-threatening acute illness or injury. The MH1813 handles approximately 1 million calls per year, is an integrated part of the Emergency Medical Services and has incorporated the Out-Of-Hours (OOH) services. All acute non-life-threatening contacts to the health care system within the region are pre-assessed by a registered nurse or physician, who triage the caller to either advice and self-care, own general practitioner (GP), clinic consultation at an emergency department, home visit, hospital admission, or dispatch of an ambulance. Approximately 40% are triaged to either self-care or their own GP. Triage is guided by a criterion-based electronic triage tool, which is a detailed instruction based on symptoms and symptom intensity that guides the call handler in questions and responses. The triage tool used at the medical helpline is developed locally and has not been validated. The population for this study consisted of callers to the MH1813 for perceived non-life-threatening illnesses/injuries.

### Participants

The data collection was conducted by telephone from January 24th to February 9th, 2017 and linked to the patient’s personal identification number (PIN) making linkage with registers possible. We included all patients calling during the data collection period, in case of several contacts we only used the first call. The choice of using the first call was based on the clinical perspective of possible under-triage. Exclusion criteria were refusal to participate, call by bystander (not relative or close friend), missing information on DOW and calls from persons without permanent Danish residency since they cannot be followed in registers. This study was planned and performed on the same study cohort used for a randomized controlled trial (RCT) [26]. The aim of the RCT was to investigate the effect on triage of the call handlers’ awareness of the callers DOW, and found no difference in proportion of callers triaged to a face-to-face consultation between intervention group and control group. The lack of effect on DOW on triage response was likely due to the fact that the call handlers did not pay attention to the displayed DOW [26].

### Data sources and variables

Data was derived from four different sources.

#### Registration at the MH1813

The internal data-registration has an incorporated triage tool where contact and patient related variables are registered. We collected information on reason for calling and triage response (see below).

*The Danish Civil Registration System* includes all permanent residents in Denmark [27]. Information on date of birth, gender, migration and mortality was obtained from the register.

*Danish National Patient Register* is a nationwide register that holds information on all in-hospital contacts including diagnoses and procedures since 1977 and emergency room and out-patient contacts since 1995. Information on date and time for hospital admission along with discharge and primary diagnosis were collected [28].

#### Survey data

Data was collected electronically using a message on the telephone while callers were waiting in line. Callers who agreed to participate were asked to state if they were the patient themselves or a relative/friend. Hereafter, callers were asked to rate their DOW by answering the question: “How worried would you say you are about the situation you are calling about on a scale from 1 to 5, where 1 is minimal worry and 5 is maximum worry?”

### Covariates

Triage response was categorized into three groups: Telephone consultation (advice of self-care, referral to own GP), face-to-face consultation (primary care health professional, consultation at emergency department, direct hospital admission, and ambulance dispatch), and other (e.g. prescription of medicine). Age was categorized (0–5, 6–17, 18–65, 66+ years). Co-morbidity was estimated by the Charlson comorbidity index [29, 30] including diagnoses for all hospital contacts up to 10 years before the call, and then grouped into no co-morbidity (score 0), low co-morbidity (score 1) and moderate or high co-morbidity (score 2+). Reason for calling was categorized as somatic illness, somatic injury, psychiatric illness and other. Caller status was divided into the patient themselves or relative/friend. The variables of age and co-morbidity were collected as continuous data, which resulted in violation of goodness of fit. Therefore, the categorized variables were used for adjusting the models.

### Outcome measures

The outcome measure was hospital admission defined as hospital stay ≥24 h starting within 48 h after the call to the medical helpline.

### Statistical methods

The association between the five-point DOW and hospital admission was evaluated with three logistic regression models: A crude model, an age-gender adjusted model and a fully adjusted model including age, gender, co-morbidity, reason for calling and caller status. The goodness of fit for the regression models was assessed by the Hosmer and Lemenshow test, which did not indicate violation of model fit for the crude analysis (*p* = 1.00), the age-and gender adjusted analysis (*p* = 0.58) and the fully-adjusted model (*p* = 0.15)) Odds ratios (ORs) were estimated with DOW 1 as reference.

Results were reported as ORs with the corresponding 95% confidence intervals (95%CI). A significance level below 0.05 was considered significant. Data was analyzed using SAS enterprise 7.12.

A sensitivity analysis was carried out, including the last call in the crude model rather than the first call. Non-response bias was assessed with information on gender, age, reason for contact and triage response for all calls during the data collection period compared to the study population. Data from the latter was collected from administrative data (Appendix).

## Results

### Population

**Fig. 1:**
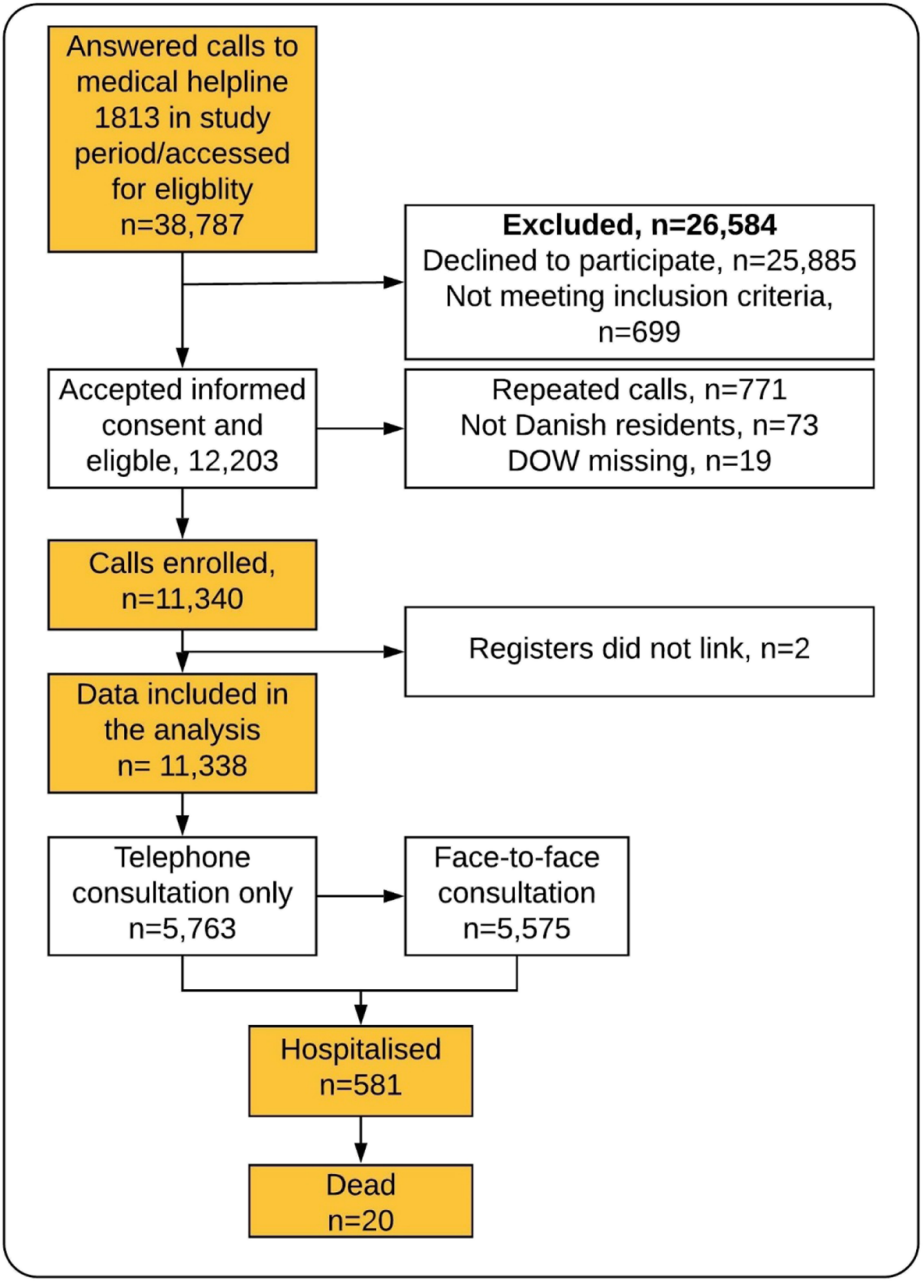
Flowchart of included calls. Triage outcome (consultation vs. face-to-face consultation) is as registered in the Danish National Patient Register – the absence of an entry in the Danish National Patient Register indicates a contact that has received telephone consultation only

A total of 38,787 patients made a call to the MH1813 during the data collection period (Fig. 1). Of these, 12,902 (33%) agreed to participate. As 699 calls were made by a person not being the patient or a relative/friend of the patient, 12,203 calls were eligible for the study. However, 19 calls were excluded due to missing DOW (the phone was answered by a call handler before the questionnaire was ended), 73 calls were from persons without permanent residency and 771 calls were repeated calls, where only the first call was included. Two calls could not be linked to the National Patient Register. Thus, 11,338 calls were included in the final analysis.

Half of the participants were female (54%) and the median age was 30.5 years (Table 1). A total of 1073 callers (9%) reported minimal worry (DOW = 1) and 2396 (21%) a DOW = 2, while the largest group reported DOW = 3 (36%). A total of 2283 (20%) reported a DOW = 4 and 1500 (13%) were maximum worried (DOW = 5).

**Table 1:** Descriptive information of study population

**Gender, n**	11,340
Female, n (%)	6137 (54.1%)
Male, n (%)	5203 (45.9%)
**Age, n (%)**	
Mean, **(**SD)	30.5 (25.5)
0–5 years	2608 (23.0%)
6–17 years	1949 (17.2%)
18–65 years	5317 (46.9%)
66+ years	1466 (12.9%)
**DOW, n (%)**	
1	1073 (9.5%)
2	2396 (21.1%)
3	4088 (36.1%)
4	2283 (20.1%)
5	1500 (13.2%)
**Reason for calling, n (%)**	
Somatic illness	6119 (54.0%)
Somatic injury	2048 (18.1%)
Psychiatric illness	51 (0.5%)
Other	444 (3.9%)
Not registered	2678 (23.6%)
**Triage outcome, n (%)**	
Telephone consultation (not seen)	5763 (50.8%)
Face-to-face consultation	5575 (49.2%)
Admitted to hospital^b^	581 (5.2%)
Missing	2
**Primary ICD-10 diagnosis in face-to-face consultation, n (%)**^a^	**5575**
Injury or external cause of morbidity	1749 (31.4%)
Unclear symptoms and other factors influencing health status	1055 (18.9%)
Respiratory	979 (17.6%)
Infections	460 (8.3%)
Eye and ear	307 (5.5%)
Urogenital	242 (4.3%)
Digestive	205 (3.7%)
Musculoskeletal	180 (3.2%)
Skin	135 (2.4%)
Circulatory	122 (2.2%)
Psychiatric illness	28 (0.5%)
Other	113 (2.0%)
Missing	2
**Comorbidity score, n (%)**	
0	9202 (81.2%)
1	1133 (10.0%)
2+	1005 (8.9%)

^a^Other: nervous system, pregnancy and birth, perinatal illness, malformation and anomalies, neoplasm, blood and blood forming organs, endocrine diseases

^b^Hospital admission is defined as having occupied a hospital bed ≥24 h. This variable is calculated by subtracting the in-date-and time from out-date-and time

The main reason for contact was somatic illness/injury (72%), whereas psychiatric illness contributed with less than 1, 4% were miscellaneous questions e.g. answers to blood tests, other guidance, or case summary after home visits and 24% had a missing reason for calling. A total of 49.2% (*n* = 5575) of the calls ended with an acute care contact registered in NPR (hospital face-to-face consultation).

### Hospital admission

Of 11,338 telephone contacts, 581 (5.2%) persons were admitted to the hospital (Fig. 1) within 48 h of calling. Maximum DOW (DOW = 5) had a crude OR for being admitted to the hospital of 6.1 (95%CI 3.9 to 9.6) compared to those with minimum DOW (Table 2). Callers with DOW = 4 had an OR of 4.7 (95%CI 3.0 to 7.3), DOW = 3 an OR of 1.8 (95%CI 1.1 to 2.8), and DOW = 2 an OR of 0.8 (95%CI 0.4 to 1.3). Adjusting for age and gender, callers with a DOW = 5 had an OR of 4.0 (95%CI 2.5 to 6.3), DOW = 4 had an OR 3.7 (95%CI 2.4 to 5.8), DOW = 3 OR 1.7 (95%CI 1.1 to 2.6), DOW = 2 OR 0.8 (95%CI 0.5 to 1.4) When adjusting for age, gender, co-morbidity, reason for contact, and caller status, the estimates changed slightly compared to the age and gender adjusted model, DOW = 5 OR 3.1 (95% CI 2.0 to 5.0), DOW = 4 OR 3.2 (95% CI 2.0 to 5.0), DOW = 3 OR 1.6 (95% CI 1.0 to 2.6), DOW = 2 OR 0.8 (95% CI 0.5 to 1.4) compared to DOW = 1.

**Table 2:**
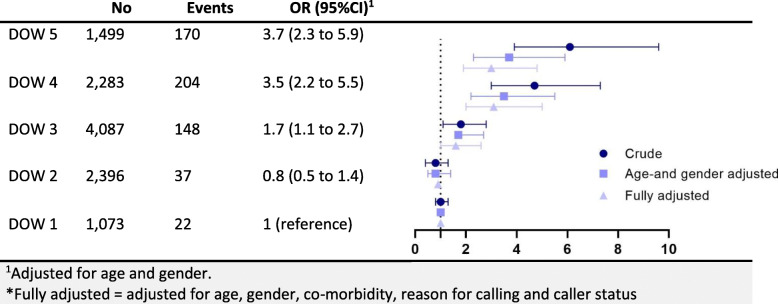
Odds ratio for hospital admission (OR, 95% CI, forest plot)

### Sensitivity and non-response analyses

The sensitivity analysis of using the last calls instead of the first calls barely changed the estimates.

Non-response bias analysis showed that there was no difference between all callers and the study population for age, gender and triage response (Appendix).

## Discussion

### Main findings

This study tested the hypothesis that callers’ subjective feeling of illness severity measured as DOW was associated with hospital admission within 48 h after a call to a medical helpline. We found a strong dose-response association between callers’ DOW and hospital admission adjusted for age, gender, co-morbidity, reason for calling and caller status where callers with a maximum DOW had three-fold increased odds of hospital admission compared to those with a minimum DOW.

### Strengths and limitations

The study benefits from the longitudinal design with complete follow-up in the high quality Danish registers. Convergent validity of the scale was seen in the dose-response of DOW and association with hospital admission [31]. A major strength of the study was the electronic setup where recall bias was bypassed; however, there might still be response (e.g. malingering) and construct bias. Malingering could have affected the results towards a higher DOW but less hospital admission, which would decrease the strength of the association. One important limitation of the present study was the fact that 25,584 (66%) callers refused to participate, which could introduce selection bias [32]. An assessment of non-respondents showed that non-respondents were similar to participants with regard to age, gender and triage response, but selection bias cannot be ruled out. Yet, very worried callers might not have wished to participate, however this fraction is supposedly limited as only a small low number of callers (< 4%) used an emergency access button to bypass the telephone waiting line in a similar study [33]. Moreover, potential severe illnesses which were treated or refuted in less than 24 h were not included in the endpoint, which might have drawn the results in the direction of an underestimation of the association between DOW and hospital admission. It has been discussed whether hospital admission is a good proxy for true illness severity and while no single outcome captures this concept it is suggested to use the methodology of diagnostic research where the outcome is dichotomized into the presence of illness yes/no [34]. We could have included endpoints such as: radiology, various treatments (e.g. inhalation, ECG), and prescriptions, however these endpoints could introduce bias in relation to differences in frequency in illness/injuries. Therefore, we chose to use hospital admission defined as hospital stay ≥24 h as the proxy for illness severity. In a large register study on the scope of the OOH from Denmark (*n* = 7810 contacts) the researchers found that *n* = 102 (1.3%) were calls regarding chest pain and 29.4% of these were admitted directly to hospital [35]. These and other potential severe illnesses might have been discharged within 24 h and might therefore not contribute to the outcome measure of hospitalization ≥24 h. This study did not address the predictive value (sensitivity and specificity) of DOW as the study design did not allow this analysis. Therefore, we cannot conclude that DOW is suited to increase precision in telephone triage. Lastly, we did not detect a significant difference between levels of DOW and odds for hospitalization, however a dose-response trend was present, although the estimates might suffer from a lack of power or callers’ discriminative ability, especially between DOW levels 4 and 5. We used the same study population in the present study and the RCT investigating the effect of DOW on triage response. In the RCT, we tested if call handlers’ awareness of DOW affected the triage response. We did not detect a difference in triage response in the RCT. Moreover, a qualitative investigation showed that the addition of DOW was to a large extent “missed” by call handlers due to several structural issues [26]. Therefore, we do not suspect the results of this study to be affected by the intervention.

### Comparison with the literature

The results from this study show that the measure of the subjective feeling of worry in telephone triage provides an indication of risk of hospital admission. Studies regarding the common-sense model and self-rated health can be used to understand how and why self-evaluation of a condition predicts health outcomes. Self-rated health is an independent predictor of 5-year mortality, people rating their health to be bad compared to good have 1.5 to 3.0 higher odds for mortality [19, 20]. Self-rated health is an inclusive, usually five-point rating, that is thought to provide individual but contextual relevant information on bodily conditions, sensations, emotions and feelings [22, 23]. However, whereas self-rated health predicts acute hospital admission due to chronic illness, this association has not been found in acute illness/injury [36, 37]. We suggest that DOW might be associated with self-assessed illness severity in the short time span, while self-rated health evaluates chronicity and predicts mortality in the longer run.

In a meta-analysis of The Brief Illness Perception Questionnaire, which is a questionnaire based on the common sense model, the results indicate that higher concern (emotional response) predicts worse outcome in illness [14]. In this study, risk of hospital admission is strongly associated with the level of DOW. However, strong illness identity has been shown to be positively associated with emotional coping strategies [24], and one could argue that patients who openly show their worry are more inclined to receive treatment/hospital admission. There is a relation between the conceptual theory behind the common-sense model and DOW. Secondary analysis of data from a feasibility study on DOW found that the caller with a strong illness identity, short illness duration, a clear cause and solution for cure and control were more likely to present a low DOW [38]. In the current study, we present a strong association between DOW and hospital admission and thus established the connection between illness representation, DOW and outcome.

Implementation of DOW in telephone triage could benefit as an addition to the existing criterion-based triage tools in clinical decision-making by introducing early patient participation in emergency care [39–41]. Traditionally, emergency medicine has been dominated by the acute-care paradigm, where the power asymmetry between provider and patient is evident [42, 43]. There is a general sparsity of studies on patient participation in acute and emergency medicine setting [44]. The implementation of DOW could initiate patient centeredness and patient participation, and in doing so take a step in the direction of a paradigm shift in acute and emergency care. One of the few studies on patient participation in emergency care hypothesized that patients’ autonomy in decision-making was inversely associated to higher level of urgency. The prospective study found that patients indeed wished to be involved in decision-making regardless of level of acuity (*p* = 0.41) [45]. The emotional feeling of worry is a catalyst for help-seeking, however, this is rarely touched upon in telephone triage [17, 46]. A Danish study of 200 audio-recorded telephone triage conversations found that callers were invited to express their emotional state in less than 2% of the calls [10]. In an analysis of malpractice claim cases the authors found that a failure to respond to the callers’ concern, closed biomedical questions and less information sharing characterized malpractice claims in cases compared to matched controls [15, 16, 47]. In a feasibility study on DOW we found that the question of “how worried are you about the situation, you’re calling in about on a scale from 1 to 5” probed more medical information sharing, and could facilitate an investigation into the callers worry with e.g.: “you say you score four in worry, why is that?” [18]. The strength of this scale lies in the simplicity and a systematic implementation of DOW in triage could increase patient-centered communication and emphasize the focus on the patient’s psychosocial resources in acute illness/injury, by simply asking the question of DOW and asking why DOW was rated to the specific number. The implementation of DOW in triage could also serve as a tool in the clinical decision-making process. It has been shown that the stressful environment of telephone helplines causes increased conservative triage response [48], therefore the integration of DOW in triage might also benefit resource allocation by creating a mental decoupling within the health professional [41] . Whether DOW could be used to triage the patients to higher or lower urgency remains to be evaluated.

#### Recommendations for future practice

The findings in this study are most likely not limited to medical helpline telephone consultation but could be applied to all health care settings with an acute patient influx that uses telephone triage. The impact of introducing DOW in triage should be evaluated by the stake holders (callers and call handlers), and an assessment of the effect on patient involvement and patient outcome and safety would be required. Barriers for implementing DOW should be explored as well as the effect on triage in both telephone triage as well as in physical triage. Moreover, studies with less strict endpoints like e.g. prescriptions, shorter hospital stay, and treatments could further validate the findings of this study.

## Conclusion

Callers’ subjective feeling of illness severity measured as DOW was strongly associated with hospital admission within 48 h after contacting a medical helpline. A high DOW was associated with higher odds for hospital admission by six-fold in crude analysis and by three -fold in the age, gender, co-morbidity, reason for contact, and caller status adjusted analysis. DOW could be included in the existing clinical decision tools and support clinical decision making by telephone. An integration of the DOW scale in acute health care telephone consultations might help the callers to express their feelings and emotions motivating their contact to an acute care telephone helpline.

## Appendix

**Table 3:** Descriptive of the full population with a valid personal identification number in the data collection period compared to the study population

	All calls, *n* = 38,787 (100%)	Study population, *n* = 11,340 (33%)
**Gender**, n (%)		
Female	21,248 (54.8%)	6137 (54.1%)
Male	17,538 (45.22%)	5203 (45.9%)
**Age**, n (%)		
0–5 years	8994 (23.2%)	2608 (23.0%)
6–18 years	6236 (16.7%)	1949 (17.2%)
19–65 years	19,270 (49.7%)	5317 (46.9%)
66+ years	4287 (11.1%)	1466 (12.9%)
**DOW**, n (%)		
1	N/A	1073 (9.5%)
2		2396 (21.1%)
3		4088 (36.1%)
4		2283 (20.1%)
5		1500 (13.2%)
**Self-rated health**, n (%)		
1, very good	N/A	2120 (18.9%)
2, good		2750 (24.5%)
3, fair		2462 (21.9%)
4, bad		2222 (19.8%)
5, very bad		1680 (15.0%)
Missing		106
**Reason for calling, n (%)**		
Somatic illness	20,726 (53.4%)	6119 (54.0%)
Somatic injury	6554 (16.9%)	2048 (18.1%)
Psychiatric illness	217 (0.6%)	51 (0.5%)
Other	2017 (5.2%)	444 (3.9%)
Missing	9273 (23.9%)	2678 (23.6%)
**Triage response**, n (%)		
Telephone consultation	16,980 (43.8%)	4993 (44%)
Face-to-face consultation	18,781 (48.4%)	5649 (50%)
Other^a^	3026 (7.8%)	771 (7%)
Missing	0	0

^a^ triage response “other” e.g. other guidance, answer on blood test, case summary after home visit, request for prescription

## Abbreviations

MH1813: Medical helpline 1813
PIN: Personal Identification Number
